# Beyond Passage Numbers: How Culture Conditions and Population-Doubling Metrics Reporting Shape the Quality of Umbilical Cord-Derived MSCs and Extracellular Vesicles

**DOI:** 10.3390/ijms27125254

**Published:** 2026-06-10

**Authors:** Carolina Quintero-Gil, Wendy V. Jaraba-Álvarez, Catalina Machuca-Acevedo, Víctor Gómez, Karolynn Halpert, Dianny Jiménez, Hector Ortega-Arellano

**Affiliations:** 1 BioXtech, Medellín 050021, Colombia; carolina.quintero@bioxcellerator.com (C.Q.-G.); catalina.machuca@bioxcellerator.com (C.M.-A.); karolynn@bioxcellerator.com (K.H.); 2 BioXcellerator, Medellín 050021, Colombia; wendy.jaraba@bioxcellerator.com (W.V.J.-Á.); victor.gomez@bioxcellerator.com (V.G.); 3 Nave Biotech, Brasilia 70200-004, Brazil; dianny@navebiotech.com

**Keywords:** mesenchymal stromal cells, umbilical cord-derived MSCs, population-doubling level, passage number, cell expansion kinetics, replicative senescence, cell manufacturing, regenerative medicine

## Abstract

Mesenchymal stromal cells (MSCs) are central to regenerative medicine and advanced therapies. However, the absence of consensus on reporting kinetic parameters, such as population doubling level (PDL), population doubling time (PDT), and the reliance on passage number alone obscures biological age and manufacturing history, and limits correlation of potency with expansion dynamics. Here, we clarify the distinctions among passages, PDL, PDT, and replication rate; we synthesize evidence that identical passage numbers can conceal multifold differences in cumulative doublings, with downstream effects on transcriptomic stability, and immunomodulatory performance. We further highlight culture determinants, oxygen tension, seeding density, media formulation, surface/bioreactor systems, and early niche mimetic stimuli, that shape proliferative kinetics and cellular aging trajectories in WJ-MSCs. Critically, we propose extracellular vesicles (EVs) as sensitive functional readouts of bioprocess stress and expansion history: EV quantity can increase while functional bioactivity declines, and EV miRNA cargo captures cell state programs not evident from minimal identity markers. To address these gaps, we recommend a reporting framework that incorporates: (1) culture conditions, (2) passage number and PDL at harvest, and (3) functional consequences of expansion. Adopting kinetic metrics beyond passage number will harmonize data capture and enable pooled analyses, accelerating clinical translation while safeguarding patient outcomes.

## 1. Introduction

**Figure 1:**
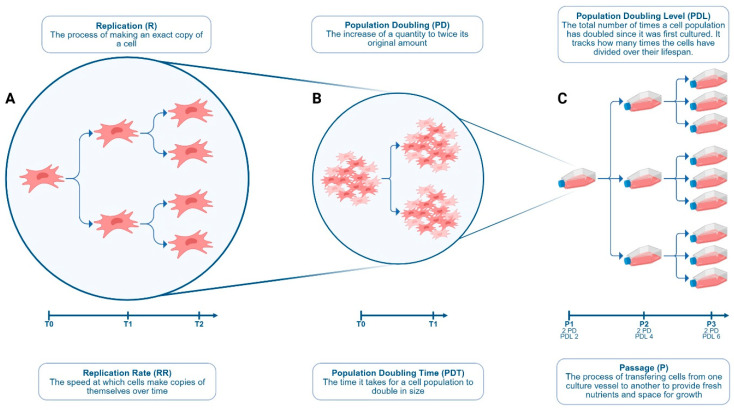
Overview of key concepts in cell expansion. The RR of cells determines how quickly they divide, influencing the PDT, or how long it takes for the total population to double. Each P involves transferring cells to fresh conditions, allowing for further growth, while the PDL tracks the total number of times the population has doubled over its lifetime. Replication rate and PDT describe time-related growth dynamics, whereas replication events, PD, and PDL indicate how many times the cells or the population have proliferated. P reflects sequential culture events rather than time or growth units. (**A**) Replication vs. Replication Rate. (**B**) PD vs. PDT. (**C**) PDL vs. P. R—Replication, RR—Replication Rate, PD—Population Doubling, PDT—Population Doubling Time, PDL—Population Doubling Level, P—Passage.

Mesenchymal stromal cells (MSCs) can be isolated from multiple tissue sources, including bone marrow, adipose tissue, and perinatal tissues such as the umbilical cord, and are widely used in regenerative medicine due to their immunomodulatory and differentiation capacities. Among these, Wharton’s jelly-derived MSCs (WJ-MSCs) have gained particular interest due to their neonatal origin, high proliferative capacity, and strong immunomodulatory properties, as well as their accessibility and favorable ethical profile, supporting their use in both research and clinical applications [1,2]. However, the lack of standardized reporting on key parameters such as passage number and population doubling level (PDL) has led to inconsistencies in experimental reproducibility and clinical outcomes [1,3]. Core concepts remain inconsistently defined and are not systematically reported across studies. In particular, passage number should be clearly defined as the number of times cells are subcultured, with explicit reporting of the starting point (commonly referred to as passage 0, corresponding to the end of primary culture following initial isolation). This distinction is essential, as variability in defining passage 0 introduces ambiguity when comparing expansion histories between laboratories. In this context, population doubling (PD) refers to a single event in which the cell population doubles, while the population doubling level (PDL) quantifies the cumulative number of such doublings since initial seeding (Figure 1). Complementarily, population doubling time (PDT) describes the time required for one doubling cycle and reflects proliferation kinetics under specific culture conditions. Although passage number and PDL are often used interchangeably to estimate cellular age, they are not equivalent and can vary significantly depending on seeding density, culture conditions, and cell source [4]. The absence of standardized definitions and reporting of these parameters limits data comparability and interpretation, ultimately contributing to variability in preclinical results and to the inconsistent clinical outcomes observed in MSC-based therapies.

Another important concept, equally relevant to passage number and population doubling level (PDL), is the replication rate (RR). Although these parameters are all related to cellular proliferation, they represent distinct metrics with specific implications for the characterization and quality control of cell cultures. Replication rate refers to the speed at which individual cells divide. It can be expressed as the number of divisions per hour or day, or as the percentage of cells actively engaged in specific phases of the cell cycle, particularly the S phase. RR is commonly assessed using proliferation markers such as Ki-67 or BrdU, which provide insights into the mitotic dynamics of actively cycling cell populations [5]. Together, these metrics are complementary; PDL enables tracking the cumulative replicative history of a cell population, while RR offers a snapshot of its current proliferative activity. The combined use of both parameters enhances the functional characterization of MSCs and strengthens traceability in therapeutic and translational applications, contributing to more robust and reproducible cell-based interventions [5].

Recent studies have shown that mesenchymal stromal cells (MSCs) undergo progressive transcriptomic and functional changes as they accumulate passages and population doublings, often preceding the appearance of classical markers of senescence [1]. These changes can adversely affect proliferation, differentiation capacity, immunomodulatory function, and the secretion rate and bioactivity of extracellular vesicles (EVs), thereby influencing therapeutic performance [2,6]. This process is particularly relevant given that extensive culture expansion is necessary to obtain clinically relevant cell numbers [7], making some degree of replicative aging unavoidable. Consequently, early passage cultures are often favored for therapeutic applications; however, what constitutes an “early” passage is not absolute and depends on both the tissue source and the expansion strategy, especially seeding density and growth kinetics, which directly shape cumulative PDL. Despite these dependencies, many clinical trials fail to report passage number or PDL, limiting traceability, reproducibility, and cross-study comparability [8].

This article explores the current literature on MSCs sources and isolation methods, the impact of passage and PDL on experimental outcomes, and the molecular pathways involved in cellular aging. We argue for the inclusion of passage and PDL in reporting guidelines and propose a framework for assessing cellular age in MSC-based interventions [9].

## 2. Conceptual Framework for Cellular Age Metrics

### 2.1. Impact of Culture Conditions and Technical Parameters on Cell Age

#### 2.1.1. Isolation Methods and Passage Establishment

Culture conditions are particularly relevant for maintaining MSC potency. The most common method for isolating WJ-MSCs is enzymatic digestion; this technique has been demonstrated by Aung et al. [8] to yield cells with consistent expression of canonical MSC markers (CD73, CD90, CD105) and trilineage differentiation potential. However, the choice of isolation method and subsequent culture conditions can markedly influence cell behavior and downstream applications.

In addition to enzymatic digestion, alternative non-enzymatic methods, such as the explant method and free tissue culture, have gained relevance due to their simplicity, reduced manipulation, and lower contamination risk. In the explant technique, Wharton’s jelly tissue is fragmented into small pieces (approximately 2–3 mm) and placed directly onto pre-treated culture surfaces. Under appropriate supplemented media conditions, MSCs gradually migrate from the tissue and adhere to the culture surface. This approach minimizes the enzymatic stress associated with digestion protocols, better preserving the extracellular matrix, and supporting higher cell viability while maintaining key phenotypic characteristics [10,11]. Similarly, free tissue culture methods avoid the use of bacterial or animal-derived enzymes, reducing the risk of endotoxin contamination, and increasing suitability for clinical applications [12]. Although non-enzymatic approaches may initially yield lower cell numbers, the subsequent expansion phase provides advantages when high cell quality is required with minimal manipulation.

In cell culture, the concept of cell passage starts at this stage, and refers to the process of transferring cells from one culture vessel to another to maintain optimal growth conditions and prevent over-confluence. Each transfer is designated as a passage, numerically labeled as P0, P1, P2, and so forth. Understanding the biological and functional distinctions between P0 (primary culture) and P1 (first passage) is essential for standardizing MSC isolation and expansion protocols, particularly for MSCs that will be used in clinical and translational research.

P0, or primary culture, represents the initial phase following the isolation of MSCs from tissue sources such as bone marrow, adipose tissue, or Wharton’s Jelly. At this stage, the culture is typically heterogeneous, containing a mixture of stromal, endothelial, hematopoietic, and other non-MSC populations. During P0, MSCs begin to adhere to the culture surface and proliferate, forming fibroblast-like colonies known as colony-forming unit fibroblasts (CFU-F). This phase is critical for selecting adherent MSCs and initiating their expansion, but it is also characterized by variability in cell composition and behavior due to the presence of non-MSC populations [2,13].

The first passage (P1) signifies the point where MSCs are enzymatically detached and re-plated into new culture vessels. This step is critical because it significantly reduces cellular heterogeneity, enriching for a more uniform and defined MSC population. From P1 onward, cells typically exhibit stable expression of MSC markers (CD73, CD90, CD105), enhanced proliferative capacity, and improved viability. Functionally, P1 cells are more suitable for downstream applications such as differentiation assays, immunomodulatory studies, and therapeutic manufacturing, as they represent a more defined and purified MSC population [14].

The transition from P0 to P1 is therefore not merely procedural but reflects a critical shift in cell population dynamics, purity, and experimental reliability. Accurate documentation of this transition is essential for reproducibility and regulatory compliance in MSC-based research and clinical protocols.

#### 2.1.2. Seeding Density

**Figure 2:**
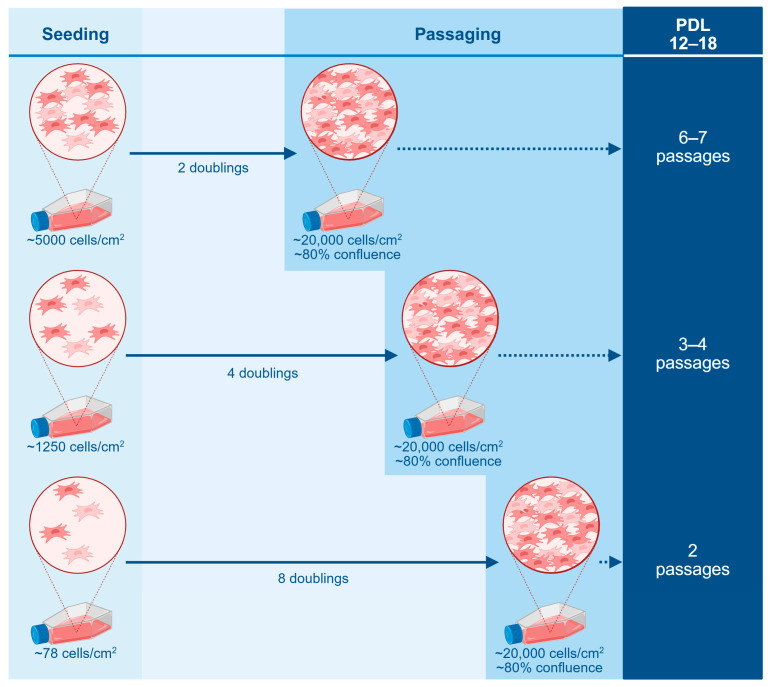
Cell density impact in PDL. Initial seeding density influences the number of population doublings achieved per passage and the total number of passages required to reach an optimal PDL range. High seeding density (~5000–7000 cells/cm^2^) results in faster confluence (~80%, ~20,000 cells/cm^2^) but yields only ~2 doublings per passage, requiring approximately 6–8 passages to reach the ideal PDL (12–18). Moderate seeding density (~1250–3000 cells/cm^2^) supports ~4 doublings per passage, reducing the expansion process to roughly 3–4 passages. Very low seeding density (~60–78 cells/cm^2^) maximizes proliferation capacity, reaching ~8 doublings per passage and achieving the target PDL range within just 2 passages [19,20]. Solid arrows indicate the number of population doublings achieved during a single passage, whereas dotted arrows indicate the approximate number of passages required to reach a cumulative PDL of 12–18.

Cell plating density is a key parameter to ensure adequate expansion rates while maintaining stemness properties, but few studies have examined the relationship between cell density (Figure 2) and the morphological and functional characteristics of mesenchymal stromal cells (MSCs). The current scientific literature suggests that plating densities, both at isolation and subculturing, can influence functional and molecular characteristics of the MSCs, and yet it is not well-standardized across laboratories. Moreover, seeding density modulates cell–cell and cell–matrix interactions, which are critical for mechanotransduction and lineage specification [15]. Venugopal et al. [16] reported that increased seeding density can override substrate stiffness effects, altering focal adhesion formation and stress fiber organization in human MSCs. These findings underscore the importance of controlling intercellular spacing to preserve the responsiveness of MSCs to biophysical cues.

Kim et al. [17] demonstrated that culturing MSCs at low cell density (50 cells/cm^2^) attenuates senescence at late passages, suggesting that cellular aging can be delayed and lifespan extended through precise regulation of cell density in vitro. Furthermore, low-density culture conditions appear to preserve the age-dependent differentiation potential of MSCs during long-term expansion. Additionally, proliferation activity was further enhanced when reactive oxygen species (ROS) production was inhibited, like the effects observed under low-density conditions [17]. These findings indicate that controlling cell density and ROS generation are effective strategies to mitigate senescence-associated changes during MSC expansion, thereby supporting improved cellular function and extended culture longevity.

In advanced applications such as MSC sheet engineering, initial seeding density determines not only the rate of confluence but also the structural integrity and paracrine potency of the resulting cell sheets. Kim et al. (2025) highlighted that variations in seeding density significantly impact sheet morphology, cytokine secretion, and therapeutic performance, emphasizing the need for standardized protocols in clinical-grade cell manufacturing [15].

While traditionally low seeding densities have been employed to promote colony-forming unit fibroblasts (CFU-F) formation and clonal selection, emerging evidence suggests that high seeding densities offer distinct advantages in terms of cell viability, proliferation efficiency, and phenotypic stability, particularly in clinical-scale manufacturing [15,18].

From a bioprocessing perspective, high seeding densities are advantageous for scaling up MSC production under Good Manufacturing Practice (GMP) conditions. They enable more efficient use of culture surfaces and media, reduce the number of required passages, and help maintain cells within early passage windows, thereby preserving their therapeutic potency. Additionally, high-density cultures have been associated with more consistent cell sheet formation and enhanced structural integrity, which are critical for tissue engineering applications [21].

#### 2.1.3. Culture Conditions and MSC Expansion Dynamics

Traditionally, MSCs have been cultured under normoxic conditions (approximately 21% O_2_). However, there has been a growing shift toward the use of hypoxic culture conditions (low oxygen levels, typically 1–5% O_2_) to better mimic the physiological microenvironment of native tissues and to precondition cells for therapeutic applications [22]. While hypoxia has been associated with improvements in parameters such as proliferation rate and cell viability [23], these effects are not consistently interpreted in the context of kinetic metrics, nor are they systematically linked to long-term outcomes such as cellular aging or functional decline. More recently, attention has also been directed toward the effects of more extreme oxygen conditions, such as hyperoxia (40–80% O_2_). Under these conditions, studies have reported a reduction in cellular proliferation and metabolic activity [24], accompanied by increased senescence and decreased viability. These effects have been associated, at least in part, with the accumulation of cell-cycle regulators such as p21 [25], suggesting that oxygen tension is a critical but underreported variable influencing MSC expansion dynamics and biological fate.

Meanwhile, ongoing advances in three-dimensional (3D) culture systems and xeno-free media formulations are further enhancing scalability and translational relevance. For example, microcarrier-based 3D cultures supplemented with human platelet lysate have shown strong performance in maintaining MSC quality attributes while improving growth and expansion efficiency [26]. Additionally, hydrogel- and scaffold-based 3D culture platforms are being developed to better mimic the native extracellular matrix, thereby improving cellular function and potentially enhancing the therapeutic efficacy of MSCs [5]. Wiese et al. cultivated Human umbilical cord perivascular cells (HUCPVCs) as a convenient and abundant source of MSCs derived from a defined region of Wharton’s jelly, in chemically defined, xeno- and serum-free media, avoiding animal-derived supplements. These cells were expanded under normoxic conditions (21% O_2_), and transcriptomic analysis revealed minimal drift up to passage 5 (PDL 27), with significant changes accumulating after passage 9 (PDL 45) [1]. This could support the use of early-passage WJ-MSCs for clinical translation, provided that PDL is measured and reported at the defined passage.

Park et al. explored the impact of mechanical stimuli, showing that pressure stimuli (2.0 PSI) under hypoxic conditions (5% O_2_) enhanced proliferation and yield of WJ-MSCs at passage 0 and after 9 days of culturing, without compromising stemness or therapeutic efficacy. Additionally, in this study it was demonstrated that applying pressure stimuli during the primary culture phase (passage 0) of WJ-MSCs significantly enhanced proliferation, enabling the production of over 20 therapeutic doses by passage 4. However, this accelerated expansion raises concerns about genomic stability, as increased proliferation rates may elevate the risk of mutation and tumorigenesis. Notably, only the initial culture phase was subjected to hypoxia or pressure, while subsequent passages were maintained under standard conditions [6]. This highlights the importance of rigorously tracking cumulative doublings and passage history to ensure the therapeutic product remains within a safe and effective biological window. Without standardized and transparent reporting of these parameters, it becomes difficult to assess the consistency, safety, and potency of MSC-based therapies.

Despite these insights, variability in isolation protocols and media formulations complicate comparisons across studies. The use of fetal bovine serum (FBS), fetal calf serum or human platelet lysate introduces batch-to-batch variability and potential immunogenicity. Chemically defined media offers a more controlled environment for MSC expansion [1], but to date, there is no consensus or specific guidelines regarding the use of culture media for MSCs intended for therapeutic applications, making it impossible to eliminate variability among manufacturers.

Within this framework of variability, studies comparing culture supplements such as human platelet lysate (hPL) and fetal bovine serum (FBS) provide concrete examples of how media composition directly influences MSC proliferation dynamics and cellular aging. In the study by Du et al. (2024), MSCs derived from two autologous sources—adipose tissue (ASCs) and bone marrow (BM-MSCs), showed significantly enhanced proliferation when cultured in hPL, as evidenced by increased cell numbers, reduced population doubling time (PDT), and upregulation of gene sets associated with cell cycle progression and DNA replication [27]. It is important to note that, as previously discussed, autologous MSCs inherently carry donor-dependent biological constraints, including age-related telomere shortening and baseline replicative pressure, which may influence their expansion behavior from the outset.

These findings suggest that while hPL supplementation enhances MSC proliferation and reduces population doubling time, it may also accelerate the accumulation of cumulative population doublings, potentially advancing replicative aging [27]. This highlights that faster growth kinetics do not necessarily equate to preserved cellular quality and provides another clear example of why reliance on a single metric is insufficient to define MSC fitness. For instance, cultures expanded under serum-containing conditions (e.g., FBS) may be reported as “low passage,” yet still exhibit a relatively high cumulative PDL due to slower proliferation rates and prolonged culture time. Without complementary metrics, such as PDL, kinetic profiling, or functional and phenotypic characterization, this discrepancy may obscure the true biological state of the cells, potentially masking cultures that are already functionally compromised and therefore suboptimal candidates for therapeutic application.

### 2.2. Passage Number and PDL in Experimental Outcomes

Passage number, which refers to the number of times cells have been sub-cultured, is often recorded as an indicator of cellular age. Cell expansion requires enzyme dissociation and cell subculture, and while the evaluation of the optimal cell confluence may vary among operators, a 70–80% confluence is recommended to be reached before detachment [28]. Passage number has traditionally tracked cellular age, but its utility is limited because it is highly dependent on specific seeding and harvest density conditions, thereby complicating comparisons across studies. Population doubling level (PDL), which refers to the total number of times the cells have doubled during in vitro culture, is, therefore, a more robust and accurate parameter to define cellular aging [29,30]. The difference between passage number and PDL is substantial and carries significant implications for cell culture standardization and therapeutic applications.

Both passage number and PDL should be essential indicators of cellular age and quality, yet they are frequently underreported in both in vitro research and clinical trials [1]. These metrics are particularly relevant when comparing MSCs from different sources, as their expansion dynamics and senescence thresholds vary. For example, bone marrow-derived MSCs (BM-MSCs) are known to exhibit donor-dependent variability and finite proliferative capacity, often entering senescence earlier than perinatal sources [31]. In contrast, WJ-MSCs demonstrate robust expansion potential and more consistent growth kinetics across donors [1]. This makes them a more reliable source for clinical-scale manufacturing.

Numerous studies comparing the expansion potential of MSCs from different sources have employed culture parameters such as passage number, cumulative population doublings (cPD), and doubling time (DT) to assess cellular aging. Consistently, WJ-MSCs demonstrate superior proliferative performance, with reported cPD values of 12.3 ± 0.7 and a population doubling time (PDT) of 21 ± 2 h, remaining stable for at least five passages. In contrast, bone marrow-derived and adipose tissue-derived MSCs (AT-MSCs) show markedly different growth kinetics. At passage 3, BM-MSCs display a significantly longer PDT (99 ± 22 h) and lower cPD (6 ± 0.5) compared to AT-MSCs, which reach 9.6 ± 0.4 cPD with a PDT of 40 ± 7 h, indicating a substantially reduced proliferative capacity in BM-MSCs. This trend persists to at least passage 9 [32].

On the other hand, Choudhery et al. compared the growth characteristics of mesenchymal stromal cells derived from WJ-MSCs and AT-MSCs, evaluating plating efficiency, maximum population doublings, population doubling time, and saturation density [33]. WJ-MSCs exhibited a significantly higher number of population doublings (33.0 ± 1.5) and a shorter doubling time (2.0 ± 0.04 days) compared to AT-MSCs (25.8 ± 0.6 and 2.7 ± 0.03 days, respectively), indicating a greater proliferative potential. However, after passage 10, WJ-MSCs displayed morphological signs of senescence and a faster decline in proliferative capacity. These findings suggest that WJ-MSCs undergo more efficient early expansion than AT-MSCs but experience an earlier loss of proliferative potential during extended culture. Collectively, these findings support that the tissue source is a key determinant of MSC proliferative behavior in culture, with WJ-MSCs representing a highly advantageous population for large-scale expansion.

Other studies have demonstrated that the phenotype and functionality of mesenchymal stromal cells deteriorate as PDL increases, negatively impacting their differentiation capacity and therapeutic potential. Niknam et al. (2024) reported a reduction in adipogenic and osteogenic differentiation in human MSCs with elevated PDL values [10]. Similarly, Le Blanc et al. (2004) observed enhanced therapeutic efficacy in early-passage MSCs compared to later passages in models of graft-versus-host disease [34]. These functional changes are increasingly understood to be linked not only to intrinsic cellular aging but also to alterations in the extracellular matrix (ECM) and cell–microenvironment interactions during in vitro expansion. As MSCs undergo successive population doublings, their ECM composition and remodeling capacity are progressively modified, affecting key signaling pathways involved in cell fate determination, adhesion, and mechanotransduction Recent evidence further supports this relationship by demonstrating that specific ECM components can actively regulate cellular aging. Lee et al. (2024) showed that tropoelastin, a soluble precursor of elastin, acts not only as a marker of youthful BM-MSCs (PDL 4 and seeded at 4000–6000 cells/cm^2^) but also as a functional modulator capable of preserving cell fitness and delaying replicative senescence [35].

Quantitatively, clonogenic capacity was shown to decline with increasing PDL regardless of culture conditions, confirming its inverse relationship with replicative aging. However, when MSCs were expanded in tropoelastin-supplemented conditions, this decline was significantly mitigated. Compared to PDL-matched controls, tropoelastin-treated MSCs exhibited an increase in colony-forming efficiency at “old cells” at PDL 7 to 12, depending on the supplementation method. Notably, while control cells showed an early 49 ± 5% reduction in clonogenic potential at PDL 7 relative to younger cells, this function was preserved under tropoelastin conditions [35].

These findings suggest that ECM dysregulation is not merely a consequence of aging but a contributing driver of the senescence process, linking replicative history (PDL) with microenvironmental alterations that ultimately impact MSC functionality.

Regarding seeding density, in the study by Aung et al. [8], WJ-MSCs were seeded at 5000 cells/cm^2^. The authors reported population doubling time (PDT), rather than population doubling level (PDL), obtaining average PDT values of 26 h at passage 1, 23 h at passage 3, and 31 h at passage 6. According to their protocol, cells at passage 6 were considered senescent, as confirmed by β-galactosidase staining and the presence of enlarged cells with altered morphology [8]. In contrast to allogeneic cells, Alves and Paiva reported that BM-MSCs reached higher cumulative population doublings across passages 1 to 5, with a culture duration ranging from 19 to 34 days, varying depending on the donor. Notably, their study also described cases of early senescence, including a patient in whom MSCs exhibited a senescent phenotype as early as passage 3 [36], highlighting that replicative limitations may not only arise during in vitro expansion but can already be intrinsic to the starting cell population. These findings are particularly relevant in autologous MSC settings, where cells are derived from patients who may already present age-related or disease-associated impairments and highlight the strong influence of tissue source and donor variability on senescence onset and replicative capacity. In MSCs derived from adult sources such as bone marrow (BM) and adipose tissue (AD), standardization of seeding density and detailed expansion reporting becomes particularly critical. Thus, the common assumption that “early passages are always preferable” may not be universally applicable, given the intrinsic variability associated with donor characteristics and expansion dynamics.

This limitation is further compounded by the ambiguity of the term “early passage” when not contextualized with quantitative growth metrics such as PDL or PDT. In the absence of standardized definitions, “early” and “late” passage classifications become arbitrary and may obscure underlying biological differences. Even within allogeneic MSC models, often assumed to be more standardized, the precise definition of expansion kinetics remains critical. For example, studies using umbilical cord–derived MSCs have classified cells at passage 3 as “early” and those at passage 15 as “late-passage”, demonstrating that late-passage cells exhibit a distinct senescent phenotype associated with activation of WNT signaling pathways. Notably, this senescence-associated phenotype was shown to be at least partially reversible through pharmacological inhibition of WNT signaling, restoring key functionalities such as trilineage differentiation potential [37]. These findings suggest that functional decline may reflect pathway-specific, potentially reversible regulatory programs rather than irreversible exhaustion. Therefore, integrating precise proliferative metrics with molecular and functional readouts is essential to accurately define MSC quality and to avoid oversimplified classifications based solely on passage number.

In addition to seeding density, source and donor variability, culture conditions such as media composition also play a significant role. In an AD-MSC model, serum-free media (SFM) conditions were compared with traditional fetal bovine serum (FBS)-supplemented cultures. Cells grown under SFM exhibited shorter and more stable PDTs compared to FBS-cultured controls at later passages (e.g., passage 15), along with a higher replication rate as measured by cumulative cell yield. In this study, a seeding density of 4000 cells/cm^2^ was used, and multipotency, senescence status, and genomic stability remained more consistent up to passage 10 under SFM conditions [37]. Altogether, these data reinforce the importance of detailed reporting of production methods and kinetic metrics, as well as the need for methodological standardization and the integration of advanced analytical approaches—such as multi-omics—for defining and releasing MSC products intended for therapeutic applications.

In regulated environments such as clinical-grade cell therapy manufacturing, relying solely on passage number is insufficient, as regulatory frameworks, including the ICH Q5D guideline, emphasize the importance of tracking population doublings throughout all stages of research, development, and production, recommending that an upper limit for PDL be established [38]. Although monitoring PDL requires more rigorous documentation and precise cell counting, it offers substantial benefits in terms of reproducibility, standardization, and quality control, ultimately ensuring the consistency, safety, and reliability of the final cell product. Despite all these, many clinical studies still fail to report essential parameters such as passage number and PDL, limiting reproducibility, traceability, and therapeutic predictability [2]. Systematic incorporation of passage and PDL reporting, already recommended across cell-manufacturing guidelines and echoed by industry analyses [4,19], would enhance transparency, support accurate assessment of MSC potency, facilitate cross-study comparisons, and ultimately advance the development of safer and more effective MSC-based therapies.

## 3. Biological Consequences of Extended Expansion in MSCs and Their Extracellular Vesicles

**Figure 3:**
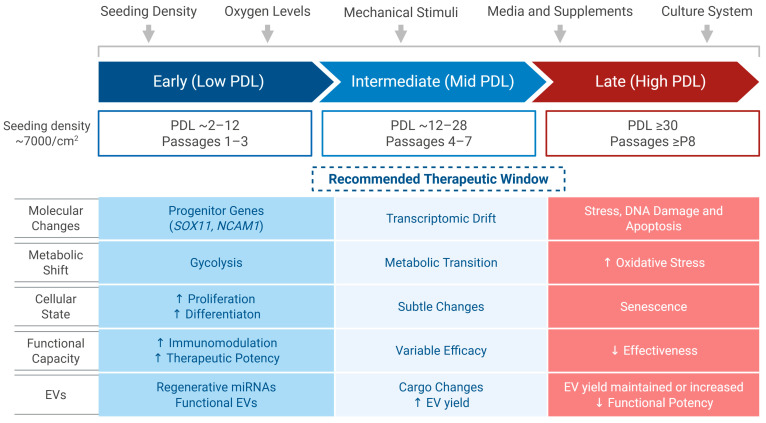
Continuum of MSC aging driven by PDL. MSC aging is depicted as a continuous process primarily governed by cumulative population doubling level (PDL), rather than passage number alone. Because seeding density directly modulates the rate of PDL accumulation, it shapes the progression of molecular, metabolic, and functional changes that define MSC state and therapeutic potential. Early-stage cells display progenitor-associated gene expression, glycolytic metabolism, and high proliferative and immunomodulatory capacity, whereas increasing PDL leads to transcriptomic drift, metabolic transition, and ultimately senescence-associated features such as DNA damage and oxidative stress. Under a high-density expansion model (7000 cells/cm^2^; 4–5 days to 80% confluence), reduced PDL accrual (~2–4 PDL per passage) extends the intermediate, therapeutically optimal window to passages 4–7, with late-stage phenotypes emerging predominantly beyond passage 8 (>30 cumulative PDL). Bold downward arrows indicate culture-related variables that can influence MSC expansion dynamics, PDL accumulation, and the progression of cellular aging during in vitro culture; ↑ increase; ↓ decrease.

Cellular aging in MSCs is a progressive and multifactorial process involving transcriptomic, metabolic, and phenotypic changes, and is mainly due to replicative senescence, described as a sequential and organized process, where cellular alterations accumulate gradually throughout in vitro expansion [39]. This structured progression suggests that senescence is not a sudden event but a continuum of biological and molecular shifts that can be anticipated and potentially mitigated through precise tracking of passage number and PDL. Understanding and identifying the early stages of this trajectory is essential to preserve the therapeutic properties of MSCs and their extracellular (EVs) and avoid the use of cells that may have already entered a decline in potency and genomic stability (Figure 3).

### 3.1. MSC Age and Mapping the Organized Course of Replicative Senescence

It is now generally accepted that MSC will begin to senesce after a certain number of cell divisions and that this is best evaluated by their PDL rather than by the number of passages or the duration of culture. As it is very difficult to evaluate the starting number of MSC in the initial culture (mixture of mononuclear cells), most labs start counting MSC cumulative population doubling at the end of the primary culture (first passage). Lechanteur et al. showed that the calculated PDL of their MSC culture ranged from 2.38 to 7.18 with a median of 4.69, which is consistent with standard MSC protocols showing 2.5–3 population doublings per passage. Moreover, they observed that PDLs are still below the PDLs correlating with occurrence of MSC senescence (PDLs from 10 to 40) [40].

In a longitudinal transcriptomic analysis of WJ-MSCs cultured in chemically defined, xeno- and serum-free media, Wiese et al. revealed that transcriptome drift begins subtly after passage 5 (PDL 27) and accelerates significantly after passage 9 (PDL 45), preceding morphological signs of senescence. This drift includes downregulation of progenitor-associated genes such as SOX11 and NCAM1, and upregulation of genes related to structural remodeling, stress response, and apoptosis. Importantly, these changes were not captured by conventional senescence markers in mid-passage cells, suggesting that transcriptomic profiling is a more sensitive indicator of cellular aging [1].

Da Silva et al. provided complementary evidence by evaluating the expression of p16 (INK4A) and p21 (CIP1/WAF1), two canonical markers of senescence. Their study showed that WJ-MSCs at passage 10 exhibited increased expression of p16 and decreased p21, consistent with cells approaching replicative arrest. Notably, p16 detection via indirect immunofluorescence was proposed as a practical and cost-effective method for identifying cultures at risk of senescence. Moreover, complementary analysis using flow cytometry revealed that p21, when assessed with antibodies capable of detecting phosphorylation states, may offer additional value for monitoring the progression toward cellular senescence, particularly in research settings where dynamic regulation of cell cycle arrest is of interest [3]. This dual-marker approach could enhance early detection of functional decline in MSC cultures and support more refined decisions in therapeutic manufacturing.

Laflaquière et al. further demonstrated that metabolic profiles of WJ-MSCs shift with passage, affecting immunosuppressive function. Their study did not use hypoxic conditions but highlighted the importance of monitoring metabolic biomarkers during expansion. The authors also added a metabolic dimension to this picture. Their dynamic metabolic modeling revealed that early-passage WJ-MSCs rely predominantly on glycolysis, while late-passage cells shift toward oxidative phosphorylation. This metabolic reprogramming is associated with increased ATP turnover, reduced proliferation, and diminished immunosuppressive function. The authors also observed that late-passage cells exhibited higher expression of genes involved in DNA damage response and cell cycle regulation, further supporting their senescent phenotype. Interestingly, BM-MSCs exhibit similar metabolic reprogramming, but in earlier passages, reinforcing the advantage of WJ-MSCs for prolonged expansion [41].

Interestingly, in HUCPVCs cultures, while some senescence-associated genes such as LEPR and FAS were upregulated in late passages (≥P9 and ≥PDL 45), others like CDKN1A and DNMT3B remained unchanged, underscoring the heterogeneity of senescence markers across MSC sources and culture conditions [1]. This variability reinforces the need for integrative approaches that combine transcriptomic, metabolic, and phenotypic data to assess cellular age.

WJ-MSCs exhibit a continuum of aging-related changes that begin at the molecular level and culminate in functional decline. Monitoring key pathways, such as WNT signaling, chromatin remodeling, and energy metabolism, alongside senescence markers like p16 and transcriptomic drift, can provide a more accurate assessment of MSC quality and therapeutic potential.

### 3.2. Extracellular Vesicles as Functional Indicators of MSC Bioprocess Performance and Expansion History

From a bioprocessing and translational manufacturing perspective, EVs derived from MSCs should be regarded as integrative functional attributes that translate cellular expansion history, including passage number, population doubling level (PDL), and culture conditions, into measurable biological outcomes related to therapeutic potency and bioprocess performance [42,43].

Accumulating evidence indicates that MSC expansion does not constitute a biologically neutral amplification step. Comparative studies demonstrate that conditions associated with cellular expansion history significantly modulate the functional bioactivity of extracellular vesicles, with a reduction in their angiogenic capacity observed even when particle number, size distribution, and expression of classical EV markers remain relatively constant [43].

At the molecular level, this expansion imprint is particularly evident in the microRNA (miRNA) profiles contained within EVs. Studies in MSCs derived from bone marrow and perinatal tissues have demonstrated that the biological state of the parental cell is reflected in exosomal miRNA cargo, with variations affecting pathways associated with cell cycle regulation, tissue repair, immune response, and programs linked to cellular aging [44,45]. Importantly, these changes may occur even when MSCs retain expression of canonical phenotypic markers (CD73, CD90, and CD105), highlighting the limitations of traditional phenotypic criteria (defined as minimal identity requirements) for predicting therapeutic potency [46].

Beyond passage number, the expansion microenvironment exerts a decisive influence on EV biogenesis and quality. Dynamic culture systems, such as stirred bioreactors, microcarrier-based platforms, and three-dimensional aggregates, consistently increase EV secretion per cell compared with conventional two-dimensional cultures [47,48,49]. However, this quantitative increase may be accompanied by functional reprogramming of the vesicular biogenesis process, associated with mechanosensitive cellular adaptations—including cytoskeletal reorganization, increased transport of multivesicular bodies (MVBs), and higher frequency of MVB–plasma membrane fusion, which reflect MSC responses to mechanical forces, shear stress, and increased biophysical demands during large-scale expansion [50]. In this context, higher EV yield does not necessarily equate to greater biological potency.

Evidence derived specifically from WJ-MSCs further reinforces the relevance of expansion history and culture conditions for EV-based applications. Across diverse experimental and translational contexts, EVs derived from WJ-MSCs have been consistently produced from early to intermediate passages, typically between P3 and P6, and evaluated in three-dimensional cartilage and intervertebral disc models, as well as injectable hydrogel systems and scalable bioreactor-based platforms, demonstrating functional biological activity under defined expansion conditions [49,51,52,53].

Quality-focused investigations have identified passage number as a key operational process variable. In WJ-MSCs, the production of small extracellular vesicles has been standardized at early passages (P3) as part of quality control frameworks for starting cellular material, consistent with the “process is the product” paradigm, positioning passage number as a controllable and reportable variable in EV biomanufacturing processes [53].

From a PDL perspective, passage number serves as an operational surrogate for cumulative proliferative history. Evidence indicates that a greater replicative history may preserve EV yield while compromising functional bioactivity, reinforcing the need to interpret quantity and potency as non-equivalent parameters [43]. Importantly, biomanufacturing studies demonstrate that EV yield per cell can be increased without raising PDL by optimizing culture conditions rather than prolonging culture lifespan. Three-dimensional architectures, microcarriers, and controlled bioreactor environments enable enhanced EV secretion while maintaining low to moderate PDLs, effectively decoupling vesicular yield from excessive population doublings associated with functional deterioration [47,48,49,54].

Importantly, emerging evidence suggests that cellular stress does not merely alter extracellular vesicle (EV) yield, but actively reshapes EV cargo composition according to the biological state of parental MSCs. Oxidative stress, replicative senescence, DNA damage responses, and mechanobiological stimuli have all been associated with selective remodeling of EV-associated miRNAs and proteins, even when conventional EV metrics such as particle concentration and canonical markers remain relatively stable [55,56].

Mechanistically, these processes involve stress-responsive pathways associated with reactive oxygen species (ROS), ESCRT-dependent trafficking, autophagy, calcium signaling, and cytoskeletal remodeling, which regulate multivesicular body transport and selective cargo loading into EVs. In addition, expansion-associated stress has been linked to activation of p53/p21 pathways, SASP-related programs, PTEN-mediated senescence signaling, and adaptive metabolic rewiring, collectively contributing to progressive alterations in EV bioactivity and therapeutic potency [57,58].

In parallel, dynamic culture systems and bioreactor expansion can activate glycolytic, autophagic, and EV-biogenesis pathways involving Rab GTPases, Alix, TSG101, and SMPD2/3, thereby increasing EV secretion while simultaneously modifying vesicular cargo profiles [42,50]. In contrast, persistent oxidative injury, serial passaging, and replicative senescence have been associated with depletion of reparative miRNAs such as miR-146a, miR-145-5p, miR-335-5p, and miR-199b-5p, alongside enrichment of inflammatory and senescence-associated cargo, ultimately impairing angiogenic, immunomodulatory, and tissue-repair functions [59,60].

Collectively, these molecular alterations may precede detectable changes in EV quantity or morphology, suggesting that EV cargo composition could represent a more sensitive indicator of MSC functional state and bioprocess-induced aging than conventional vesicle characterization parameters alone.

## 4. Discussion

The reliance on passage number as a proxy for cellular age is increasingly biologically indefensible [1,7]. Our synthesis of current evidence confirms that identical passage numbers can conceal multifold differences in cumulative doublings, directly impacting the transcriptomic and functional stability of MSCs [2,5,6]. While WJ-MSCs demonstrate superior proliferative performance—maintaining stable growth kinetics with shorter doubling times compared to bone marrow (BM-MSCs)—they are not immune to replicative drift [3,32,36].

Crucially, transcriptomic drift in WJ-MSCs has been shown to begin subtly around PDL 27 (passage 5) and accelerate significantly after PDL 45 (passage 9), often preceding the appearance of overt morphological senescence markers [1,3]. This highlights that “early passage” is an arbitrary and source-dependent label; for instance, WJ-MSCs expanded at high seeding densities accumulate far fewer doublings per passage than BM-MSCs, making a P5 WJ-MSC biologically “younger” than a P5 BM-MSC [6,32,36]. By prioritizing PDL as a robust metric, researchers can more accurately map the organized course of replicative senescence and identify the precise “therapeutic window” before functional decline begins [29,30,35]. These findings argue for adopting PDL as a release-relevant attribute in early-passage products and for explicitly reporting PDL alongside passage number in manuscripts and clinical protocols. Non-compliance with this approach may impede cross-study comparability and meta-analyses and complicates potency-to-expansion correlations central to dose justification.

Clinical-scale experiences further underscore the operational importance of ensuring that cell products remain free from senescence and metabolic alterations associated with cellular aging, while emphasizing that low cellular age, rather than simply low passage (Figure 2), represents the critical determinant of product quality. In large banking programs, MSCs from different sources met release criteria and showed acceptable safety upon infusion [40]; however, challenges recognized in clinical translation, including variable efficacy, donor heterogeneity, and manufacturing inconsistency, are aggravated by under-reporting of cellular age metrics [28]. 

Source-specific kinetics also justify routine cellular age quantification. Comparative analyses across tissue sources reveal distinct proliferation and immunomodulatory profiles, with WJ-MSCs generally exhibiting robust expansion and growth kinetics compared to adult sources yet still undergoing drift beyond defined PDL windows [32]. Properly reporting PDL enables valid comparisons and supports rational source selection for indications or manufacturing platforms. Moreover, pre-conditioning strategies, such as hypoxia or mechanical stimulation during primary culture, can accelerate proliferation and bring products to therapeutic yield at earlier passages [61,62]. Without cumulative doubling data, such acceleration cannot be evaluated for trade-offs in genomic stability or long-term potency. Park et al. showed that pressure stimuli under hypoxia enhanced WJ-MSC proliferation during P0 while maintaining stemness but emphasized the need to ensure safety as expansion is accelerated [6], a requirement most effectively addressed through systematic monitoring and reporting of population doubling level.

The translational manufacturing literature increasingly adopts GMP-compliant methods for MSCs, spanning isolation optimization, xeno-free expansion, and scale-up to pilot bioreactors. These studies highlight the importance of process parameters (e.g., hypoxic pre-conditioning, different culture supplementation, or platelet lysate concentration) on yield and quality across passages, and advocate standardized, scalable processes [2,27,30,63]. Reporting PDL throughout these workflows creates a common denominator to link process conditions with biological age and potency, easing technology transfer and regulatory review. As advanced 3D microcarrier systems and large stirred-tank bioreactors achieve multi-fold expansion to billions of cells, PDL serves as a critical control to avoid drifting past quality boundaries while meeting dose needs [24].

Historically, the criteria used to define the optimal therapeutic window for mesenchymal stromal cells have relied heavily on passage number as a surrogate marker of replicative aging. This convention emerged from heterogeneous clinical and experimental studies, primarily involving bone marrow-derived MSCs expanded from very low seeding densities [20], where early passages (commonly P3) coincided with relatively limited proliferative history. This paradigm warrants reconsideration and is why standardized reporting of PDL/PDT can mitigate these factors by enabling potency normalization across lots and trials.

In regulated GMP-compliant manufacturing and in certain regulatory contexts, including specific clinical trial approvals under authorities such as the European Medicines Agency (EMA), the quantification and reporting of population doublings are required as part of the characterization and comparability assessment of cellular products. At the same time, there is growing recognition that existing characterization guidelines may require updating to better accommodate the diversity of MSC populations and manufacturing approaches, and tracking population doublings is not merely a recommendation but a requirement [38,42]. The ICH Q5D guidelines already mandate the documentation of cell expansion and the establishment of upper PDL limits to mitigate risks associated with genomic instability and tumorigenicity [38,42,58].

Similarly, the International Society for Cellular Therapy (ISCT) has established foundational criteria for MSC identification, originally proposed in 2006 and later expanded to incorporate functional considerations such as immunomodulatory assays and potency-related evaluations under the Investigational New Drug (IND) pathway [64]. However, the ISCT has explicitly clarified that these guidelines were not intended to serve as final product release criteria. Despite this distinction, regulatory frameworks for advanced therapy medicinal products (ATMPs), along with interpretations within the literature and FDA regulatory submissions, suggest that these criteria may in practice be treated as equivalent by some stakeholders [9]. This ambiguity further underscores the need for clearer and more standardized characterization frameworks. The evolving perspective reflects a broader recognition that reliance on phenotypic identity alone is insufficient and that the lack of uniformity in characterization and reporting has contributed to inconsistencies across the field.

Notably, although current regulatory standards do not explicitly mandate the evaluation of extracellular vesicles (EVs), this represents an emerging and highly relevant area. Given that the primary mechanism of action of MSCs is widely attributed to paracrine signaling, largely mediated by EVs [9], their functional characterization may provide an additional and highly informative layer of quality assessment. Importantly, emerging evidence further reinforces and operationalizes this concept by demonstrating that EVs reflect the underlying expansion history of MSCs. From a translational standpoint, two MSC preparations harvested at similar passage numbers may produce EVs with substantially different biological activities if they differ in cumulative PDL or expansion conditions.

Any proposed passage or PDL ranges should be interpreted as context-specific rather than universally applicable, as they depend on multiple variables including cell source, donor characteristics, culture conditions, and expansion systems. In this regard, we emphasize the importance of standardized and transparent reporting, rather than prescribe fixed optimal thresholds. To support this approach, a conceptual framework for reporting MSC expansion conditions and kinetic parameters is intended to guide consistent data capture and interpretation across studies, acknowledging the multifactorial nature of MSC biology and the necessity of contextualizing expansion metrics within a broader set of functional and bioprocess variables (Table 1).

Collectively, these considerations motivate a shift beyond “passage-only” reporting toward a concise cellular age dossier for each experiment and manufactured lot: (i) culture conditions (including isolation method, culture media and supplements, oxygen levels, and seeding and harvest densities), (ii) passage number and PDL at harvest, and (iii) potency-relevant metrics (such as immunomodulatory assays and secretome or extracellular vesicle profiles) contextualized by cellular age (Table 1). This integrated framework aligns with cGMP expectations for identity, purity, and stability, and strengthens the interpretability of preclinical and clinical outcomes. The practical benefits include improved reproducibility across laboratories, facilitated pooled analyses, and more defensible dose-limiting potency assessments in relation to manufacturing history. Taken together, integrating cumulative PDL with EV-based functional profiling offers a scalable and source-independent strategy to link bioprocess conditions with true cellular age, enabling more rational definitions of therapeutic potency across diverse MSC manufacturing platforms.

## Figures and Tables

**Table 1:** Recommended Minimum Reporting Framework for MSC Expansion Studies.

Category	Minimum Parameters to Report
Cell source	Tissue source, donor information
Isolation	Isolation method, P0 definition
Culture conditions	Media, supplements, oxygen tension, culture system
Expansion metrics	Seeding density, harvest density, passage number, PDL, PDT
Basic characterization	Viability, adherence to plastic, immune phenotype *, tri-lineage differentiation (adipocytes, chondroblasts, and osteoblasts)
Additional characterization	Senescence, EV characterization, functional assays

* ISCT minimal criteria: Positive (≥95%) for surface antigen markers CD105, CD90, and CD73 while also negative (≤2%) for CD45 (pan-leukocyte), CD34 (hematopoietic and endothelial cells), CD14 or CD11b (monocytes and macrophages), CD79a or CD19 (B cells), and HLA-DR.

## Data Availability

No new data were created or analyzed in this study.

## Abbreviations

The following abbreviations are used in this manuscript:

MSCs	Mesenchymal Stromal Cells
UC-MSCs	Umbilical Cord-derived Mesenchymal Stromal Cells
PDL	Population Doubling Level
PDT	Population Doubling Time
WJ-MSCs	Wharton’s Jelly-derived Mesenchymal Stromal Cells
R	Replication
RR	Replication Rate
PD	Population Doubling
P	Passage
CFU-F	Colony-Forming Unit Fibroblasts
P0	Primary Culture
P1	First Passage
ROS	Reactive Oxygen Species
GMP	Good Manufacturing Practices
HUCPVCs	Human Umbilical Cord-derived Perivascular Cells
FBS	Fetal Bovine Serum
cPD	Cumulative Population Doubling
AT-MSCs	Adipose Tissue-derived Mesenchymal Stromal Cells
ICH	International Council for Harmonization
BM-MSCs	Bone Marrow-derived Mesenchymal Stromal Cells
ATP	Adenosine triphosphate
